# HIV-exposed infant follow-up in Mozambique: formative research findings for the design of a cluster randomized controlled trial to improve testing and ART initiation

**DOI:** 10.1186/s12913-020-5051-8

**Published:** 2020-03-18

**Authors:** Lúcia Vieira, Arlete Miloque Mahumane, Manuel Napua, Falume Chale, João Luís Manuel, Jessica Greenberg Cowan, Kenneth Sherr, Rachel R. Chapman, James T. Pfeiffer

**Affiliations:** 1 Ministry of Health, Centro de Investigação Operacional da Beira, Institute Nacional de Saúde, 1323 Correia de Brito Street, Ponta-gêa Health Center Building, Beira, Sofala Mozambique; 2 School of Nursing, University of Washington, Seattle, USA; 3 Department of Family Medicine, University of Washington, Seattle, USA; 4 Health Alliance International, Beira, Mozambique; 5 Department of Anthropology, University of Washington, Seattle, USA

**Keywords:** EID, Pediatric HIV, LTFU, Implementation science, Formative research, Mozambique

## Abstract

**Background:**

Early infant diagnosis (EID) of HIV-exposed and initiation of HIV-positive infants on anti-retroviral therapy (ART) requires a well-coordinated cascade of care. Loss-to-follow-up (LTFU) can occur at multiple steps and effective EID is impeded by human resource constraints, difficulty with patient tracking, and long waiting periods. The objective of this research was to conduct formative research to guide the development of an intervention to improve the pediatric HIV care cascade in central Mozambique. The study was conducted in Manica and Sofala Provinces where the adult HIV burden is higher than the national average. The research focused on 3 large clinics in each province, along the highly populated Beira corridor.

**Methods:**

The research was conducted in 2014 over 3 months at six facilities and consisted of 1) patient flow mapping and collection of health systems data from postpartum, child-at-risk, and ART service registries, 2) measurement of clinic waiting times, and 3) patient and health worker focus groups.

**Results:**

HIV testing and ART initiation coverage for mothers tends to be high, but EID and pediatric ART initiation are hampered by lack of patient tracking, long waiting times, and inadequate counseling to navigate the care cascade***.*** About 76% of HIV-positive infants were LTFU and did not initiate ART.

**Conclusions:**

Effective interventions to reduce LTFU in EID and improve pediatric ART initiation should focus on patient tracking, active follow-up of defaulting patients, reduction in EID turn-around times for PCR results, and initiation of ART by nurses in child-at-risk services.

**Trial registration:**

Retrospectively registered, ISRCTN67747315, July 24, 2019.

## Background

Diagnosis and care for children exposed to HIV remains a major challenge throughout the developing world [1–3]. The World Health Organization (WHO) estimated that by 2016 only 43% of HIV-exposed infants received an HIV test within the first 2 months of life, and by 2017 only 51% of HIV-positive children were receiving antiretroviral therapy (ART) [3]. HIV-positive infants should initiate life-long ART [4], but in many settings they are not retained in care and never start treatment. The catastrophic costs of failing to identify and treat HIV-positive infants early in life have been well described in multiple observational and randomized trials [5–7]. The Children with HIV Early ART (CHER) trial in South Africa found a 76% reduction in mortality for infants started on ART before 3 months of age [8]. However, without treatment, more than half of all HIV infected infants progress to AIDS and death by age two [8].

Identifying HIV-exposed infants, diagnosing HIV infection, and starting HIV-positive infants on treatment requires a well-coordinated cascade of care. The EID care and treatment cascade has proven challenging in many developing countries, including Mozambique [1, 9–14]. Loss-to-follow-up (LTFU) can occur at multiple steps and effective EID is further impeded by human resource constraints, lack of parental understanding for how to navigate care, laboratory stock-outs, and difficulty with patient tracking [9–14]. One recent meta-analysis indicated that most attrition occurs in the first 6 months of follow-up and 39% of exposed infants were not in care after 18 months, therefore interventions early in the EID cascade might have greater impact on LTFU [14]. To reach the UNAIDS 90–90-90 targets by 2020 improvements in the pediatric HIV treatment cascade are urgently needed [15].

Because of its high HIV burden, Mozambique was a priority country identified by the UNAIDS Global Plan (2011–2015) [16]. In 2012, just before the study described below was initiated, only 27% of eligible HIV-positive children in Mozambique under 14 received antiretroviral therapy [17], and rates for infants specifically were even more concerning.

Following WHO guidelines [4], dried blood spot collection was scaled up in many facilities during this period, with polymerase chain reaction (PCR) capacity available at centralized laboratories for EID. Referral to pediatric HIV care services was conducted at most health facilities in Mozambique. However high LTFU rates among HIV-positive mothers and their HIV-exposed infants before EID, and poor referral of HIV-positive infants to pediatric HIV care continued to impede service coverage. This formative research was conducted in 2014 in Manica and Sofala Provinces in central Mozambique, which were among those that had not reached their goals by 2013, in the year before the data presented here were collected, with 41 and 71% LTFU respectively [18]. Adult HIV burden was higher than the national average at 15.6 and 17.8% for women, 14.8 and 12.6% for men [19]. An estimated 18% of pregnant women were HIV-positive in 2009, based on the last major national survey conducted before this research began [20]. Under-5 mortality was estimated at 107 per 1000 live births in Manica and 83 in Sofala [21], and pediatric HIV infection alone was estimated to account for over 16% of under-5 mortality in the two provinces [22].

In 2013, “Option B+” (test and treat for pregnant women) was rolled out in the two provinces. According to health systems reports in 2013, approximately 210,000 women attended a first ANC visit in Manica and Sofala Provinces, and 197,000 were tested for HIV; 19,000 (9.6% of those tested) tested positive; 9648 initiated ART [23, 24]. Nearly 16,000 exposed infants were registered in child-at-risk services or CCR (*Consulta de Criança em Risco*). Approximately 10,210 PCR tests were conducted for infants less than 8 weeks old yielding 721 positives (7%); 8417 children were tested at 18 months yielding 796 positives (9.4%). However, data were not routinely collected at the provincial level for ART initiation among infants in CCR, creating challenges to assessing the treatment linkage for positive infants.

EID and ART coverage have improved since 2014 when the formative research data reported here were gathered, but major challenges remain. By 2017, the Ministry of Health (MOH) estimated nationally that only 66% of HIV-exposed infants received PCR testing, only 51% of infected children were receiving ART, and there was still an estimated 14% vertical transmission rate [25]. By 2018 in Manica and Sofala, among HIV-exposed infants who successfully were transferred to high risk children’s services, 77 and 76% respectively of infants under 2 months were tested, but estimated population testing coverage was only 70 and 64% [26]. There are no current data reported by province on pediatric ART coverage either as percentage of HIV-positive infants in CCR or as population coverage, but the rates are believed to still be quite low due to LTFU in referrals from CCR to ART services.

In 2018, the MOH indicated that this gap is a priority and that late registration in CCR, lab capacity and logistics, workforce shortages, and other factors were barriers to EID and linkages to ART [25]. The pediatric HIV treatment cascade policy in Mozambique has remained essentially unchanged over this period. Evidence gathered from recent years, such as the detailed formative research data reported here, is needed to guide current national policy development and inform decisions about improving EID and pediatric ART coverage in relationship to key findings from this study. The objective of this formative research was to collect data for the first phase of a two-phase approach to an intervention study. These first phase data were used to develop an intervention to improve the retention of HIV-positive mothers and their infants in the care cascade in six high volume public clinics in Manica and Sofala provinces from 2015 to 2017. The new approach was tested in the second study phase, described and reported elsewhere [27], through a clustered (facility-level) randomized controlled trial using a stepped wedge design.

## Methods

Quantitative and qualitative data were gathered to identify barriers and facilitators in the EID and treatment cascade, and solicit experiences, perceptions, and recommendations of HIV-positive mothers and health care workers to reduce LTFU to help in the design of a pilot intervention.

### Study setting and design

The formative research study included six health facilities, selected in collaboration with the Provincial Health Directorates (DPSs) of Manica and Sofala using criteria of high patient volume and a mix of urban and more rural facilities along the highly populated Beira corridor. The selected facilities included three in Sofala province (Macurungo, Munhava, and Dondo) and three in Manica (Nhamaonha, 1° de Maio, and Gondola). All are public facilities in the National Health Service that have provided the full range of PMTCT services, including HIV testing (rapid test and access to PCR test for EID, with 5 of 6 facilities shipping the samples to a referral lab), access to CD4 testing (2 of 6 sites shipped the samples to a referral lab) and ART. The post-partum clinic visits (known as CPP, *consulta pós-parto*) and child-at-risk (CCR, *consulta de criança em risco*) clinic visits were staffed by mid-level maternal-child health (MCH) nurses, and ART for infants and children was provided by physician assistants, known as *tecnicos de medicina (*referred to as *tecnicos)*, in Mozambique, and medical doctors. Data were collected to identify inefficiencies and bottlenecks in the follow up of mothers and their infants in maternity, post-partum, CCR and retention in ART services, to guide the identification of key workflow modifications, and develop an enhanced adherence and retention package to test in an intervention.

### Data collection

Data were collected from September to November 2014 at the six sites. The research consisted of 1) patient flow mapping, 2) time-motion studies, 3) collection of health systems data per clinic, and 4) focus group discussions with mothers and health staff to identify facilitators and barriers to patient flow and access. Individual interviews with health workers were used to map patient flow patterns from the maternity and CCR, to ART services at each of the target sites to produce flow diagrams [28]. To measure specific clinic waiting and consult times, researchers used a time-motion study method in which they were stationed at clinics and measured the waiting time and clinic visit duration from the moment mothers arrived at the CPP, CCR, and ART services through provider consultations [29]. Individual mothers were continually observed through their clinic visits to measure time spent waiting and in consultation. Two trained researchers followed 20 mothers to measure waiting time in CCR and 10 in pediatric ART in each of the six clinics (for a total of 120 for CCR and 60 for pediatric ART) over a full week per clinic. Two researchers also collected data over 3 months from health systems resources, such as maternity, CPP and CCR clinic registries, and ART patient charts and clinic registries. In maternities, the team collected data for number of births, HIV positives, ART status and prophylaxis. In CPP, data were collected on number of clinic visits, HIV positives, and children referred to CCR. In CCR, data were collected on the number of exposed HIV infant’s clinic visits, month of first PCR sample, PCR received in health facility, PCR results given to a mother, positive PCR tests, and ART initiation and retention over 3 months. Focus groups discussions (FGDs) were conducted at each site as described below.

### Patient and health worker perspectives

FGDs were conducted with mothers and health workers by research team members with one note taker [30]. One FGD with mothers (5–8 participants) was completed at each facility (total of six FGDs). With approval and support from health facility staff and leadership, mothers were purposively sampled and recruited through pre-existing mother-to-mother peer support groups organized for HIV-positive women already receiving HIV care and treatment at each facility. A research team member attended a regular group meeting and asked for volunteers to participate in a one-hour FGD. The goal of the FGDs was to capture consensus among the patients about what they experienced as the most important barriers and facilitators to accessing and continuing in care. The pre-existing peer groups provided a sample of mothers who had regularly visited the facility and had become more comfortable speaking about their experience with others they already knew in their groups. The FGDs used a semi-structured interview guide with open-ended questions developed to (1) assess patient experience with ANC, post-partum care, EID, and pediatric ART, (2) identify barriers and facilitators in accessing and navigating care, and (3) solicit suggestions to improve patient follow-up in care [30]. All volunteers were consented, and FGDs took place at the health facilities in private settings. Since the FGDs sought to identify broad consensus on key facilitators and barriers to care among current users, extensive individual demographic data were not requested from these groups. The focus groups were conducted by trained interview teams in Portuguese, with the occasional use of local terms as needed from local languages including Sena, Ndau, and Tewe.

Additional FGDs were conducted at each facility (six FGDs in total with 5–8 participants) with MCH nurses, counselors for those services, pharmacists, laboratory technicians, tecnicos, and medical doctors (where available) engaged in EID and pediatric HIV related activities. These FGDs used semi-structured interview guides with open-ended questions on three categories of information to: (1) understand the perceptions of the importance to follow up for the HIV-positive mothers and infants, (2) identify facilitators and barriers to retention, and (3) identify potential strategies to improve retention [30].

Since many participants preferred not to be recorded in both sets of groups, extensive detailed notes were taken by trained note-takers, and then entered into an Excel spreadsheet to analyze and identify key themes focusing on barriers and facilitators to care and follow-up, and recommendations for improvement [31]. For both sets of FGDs, the lead researchers individually coded for themes based on the three categories and then systematically compared codes to identify, discuss, and resolve code discrepancies in the final code lists [30, 31].

The research team also conducted 36 in-depth individual interviews (IDIs) with health workers (6 at 6 sites) to help explain and diagram work and patient flow, and help identify possible bottlenecks [30]. Respondents were drawn from the same health workers who participated in the FGDs including MCH nurses, tecnicos, doctors, and facility directors. In each of the IDIs, one interviewer with a note taker used a semi-structured interview guide to ask respondents to (1) describe their role in the postpartum, EID, and/or ART services process, (2) describe work and patient flow in their specific segment of those services, and (3) describe what bottlenecks and barriers they identify in patient flow and retention in their area of work. IDI’s lasted from 30 to 60 min and were conducted in Portuguese. Extensive notes were taken and used to help develop patient flow diagrams for each facility. Notes were also analyzed to identify key themes concerning bottlenecks and barriers to service delivery as described above for FGDs. All data from qualitative interviews were stored on the lead researchers’ computers in password protected folders following the IRB-approved protocol. The overall process was reviewed for data reporting using the COREQ checklist for qualitative research [32].

## Results

### Workforce

All facilities had MCH nurses trained to provide ART care for the pair (mother-child), but ART was still initiated by tecnicos or medical doctors per MoH policy. In all facilities there were community health workers, known as *activistas*, paid by international NGOs to support the facility and peer support “mother-to-mother” groups program. However, not all mother-to-mother groups were active in the sites. In all clinics, tecnicos were in charge of the clinical visits of HIV-positive patients and pharmacists for ARV medication provision. Macurungo and Nhamaonha health Centers did not have laboratories so MCH nurses drew blood and shipped samples to a referral laboratory for any other non-HIV testing needs. Munhava, 1^o^ de Maio, and Gondola Health Centers included psychologists and senior nurses.

### Work and patient flow mapping

**Fig. 1:**
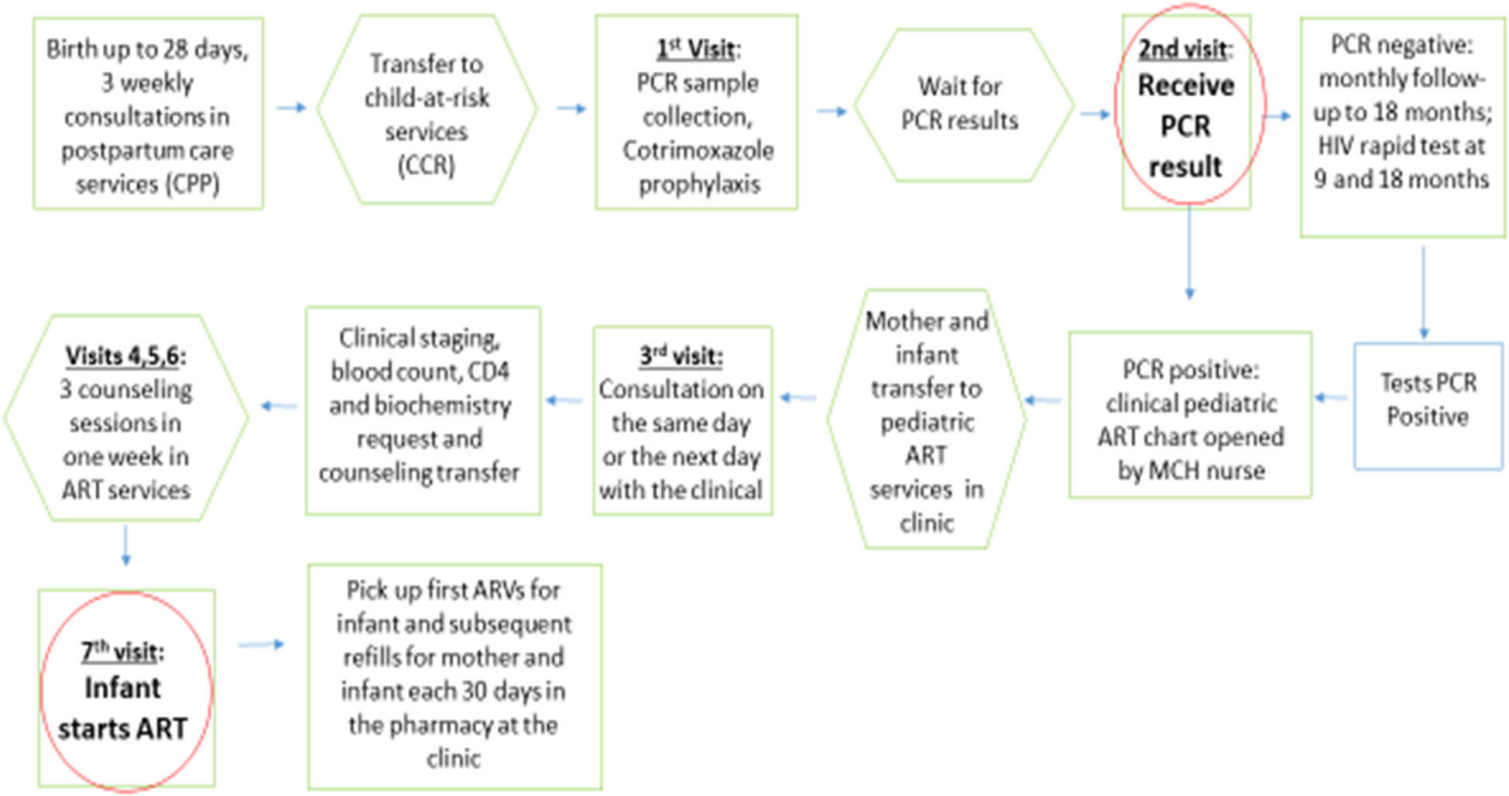
Flow Mapping of mothers with exposed or HIV positive children

The diagram in Fig. 1 depicts the flow of the mother and infant together through the testing and treatment cascade that was common to all six facilities and reflected the standard protocol. The follow-up of the mother-infant pair started at the maternity, where the mother received nevirapine syrup to administer to the infant until the 6th week of life. After 28 days, and three follow up visits at the CPP, the HIV-positive mother then continued follow up for the infant at the CCR. (Mothers who gave birth elsewhere could also seek consultations at the CPP and enter the cascade).

On her last clinic visit at the CPP, the pair was transferred to CCR, where infant blood samples were taken for PCR and non-HIV testing. (Mothers who gave birth elsewhere and did not use CPP could also arrive at CCR services through other referrals). The MCH nurse did not initiate pediatric ART in CCR, but rather opened the file and then referred both the mother and the infant to pediatric HIV services at another venue in the clinic. Once there, the child was given priority, but in principle the mother’s ART would be continued together with her infant at the same pediatric HIV clinic visit. However, there was often a long waiting period to locate the mother’s ART clinical file from the adult ART services. The child and the mother then were referred to the general pharmacy where they waited in line again to receive the ARVs, and would return in the future for regular refills each 30 days.

Data were collected from each facility to capture patient volume in the cascade for a 3 month period (see Table 1). These aggregate data indicate that the 6 facilities were achieving high coverage for initiating maternal ART, and providing ARV prophylaxis to exposed infants. However, referral rates of HIV-positive infants to CCR were generally low in all facilities.

**Table 1:** Proportion of woman followed up in Maternity and CPP in the previous 3 months (2014)

	Sofala Province			Manica Province		
**Maternity**	Munhava	Macurungo	Dondo	1_Maio	Nhamaonha	Gondola
N° of deliveries	705	296	669	601	676	727
N° of HIV+ women in maternity	170 (24%)	57 (19%)	144 (22%)	79 (13%)	77 (11%)	67 (9%)
N° of women in ART (% of those HIV+ in mat)	144 (85%)	52 (91%)	108 (75%)	69 (87%)	62 (81%)	63 (94%)
N° of infants who received HIV prophylaxis (% mothers HIV+)	145 (85%)	47 (82%)	140 (97%)	68 (86%)	53 (69%)	65 (97%)
**Post partum clinic visit**						
N° of first clinic visits	737	342	202	1127	791	1010
N° of HIV+ women at first clinic visit	233 (32%)	89 (26%)	64 (32%)	87 (8%)	29 (4%)	46 (5%)
N° of infants referred to CCR in last clinic visit (% of HIV+ mothers)	124 (53%)	55 (62%)	0	101 (53%)	20 (70%)	3 (20%)

### Clinic waiting times

Waiting times showed some variation across facilities (Table 2), but generally long waiting times for most services corroborated responses from qualitative interviews. The median duration of consultation periods for CCR only ranged from 5 to 11 min, with one outlier of 25 min in 1° de Maio. Median consult times for pediatric ART visits ranged from as little as 6 min to 36 min suggesting challenges for sufficient time to explanation and counseling by MCH nurses. A further analysis was conducted to estimate turnaround time for PCR results (Table 3). In Sofala facilities there was a mean of 26 days, with a minimum of 6 days and a maximum of 82 days and for Manica facilities it was 28 days (14 minimum and 56 maximum).

**Table 2:** Clinic waiting times

Indicator	Sofala			Manica		
	Munhava	Macurungo	Dondo	Nhamaonha	1 de Maio	Gondola
**Median waiting time at child-at-risk consultation in hours and minutes (min, max)**						
Waiting time for consultation	01:30	02:30	01:38	00:49	02:40	00:25
	(0:12, 2:29)	(1:12, 3:22)	(0:38, 3:35)	(0:03, 2:20)	(0:03, 4:48)	(0:05, 2:13)
Duration of the consultation	00:08	00:11	00:10	00:05	00:25	00:05
	(0:02, 1:11)	(0:04, 0:56)	(0:01, 0:29)	(0:01, 0:44)	(0:02, 1:02)	(0:01, 0:21)
Total time in HF	01:41	02:43	01:51	00:57	02:59	00:29
	(0:25, 2:25)	(1:21, 3:38)	(0:54, 3:45)	(0:22, 2:23)	(0:37, 4:57)	(0:08, 2:28)
**Median waiting time at pediatric ART consultation (min, max)**						
Waiting time for consultation	02:19	01:29	01:09	01:29	01:15	01:23
	(1:15, 4:56)	(1:03, 3:49)	(0:22, 3:50)	(0:47, 3:39)	(0:34, 2:27)	(0:22, 1:45)
Duration of the consultation	00:14	00:21	00:20	00:36	00:06	00:22
	(0:07, 0:28)	(0:02, 0:55)	(0:12, 0:38)	(0:02, 1: 26)	(0:03, 0:09)	(0:11, 0:42)
Total time in HF	02:35	01:53	01:30	02:00	01:19	01:40
	(1:43, 5:06)	(1:29, 3:58)	(0:42, 4:13)	(1:39, 4:14)	(0:38, 2:42)	(0:50, 2:04)

**Table 3:** Turnaround time for PCR results

	Sofala	Manica
**Turnaround time for PCR results in days, mean (min; max)**		
Time between the collection of the child’s blood sample, sending and returning results of the results in the HF	26 (6;82)	28 (14;56)
Time between the availability of the results in the HF and delivery to the mother or guardian	24 (1;99)	21 (1;120)

### Treatment cascade LTFU

**Fig. 2:**
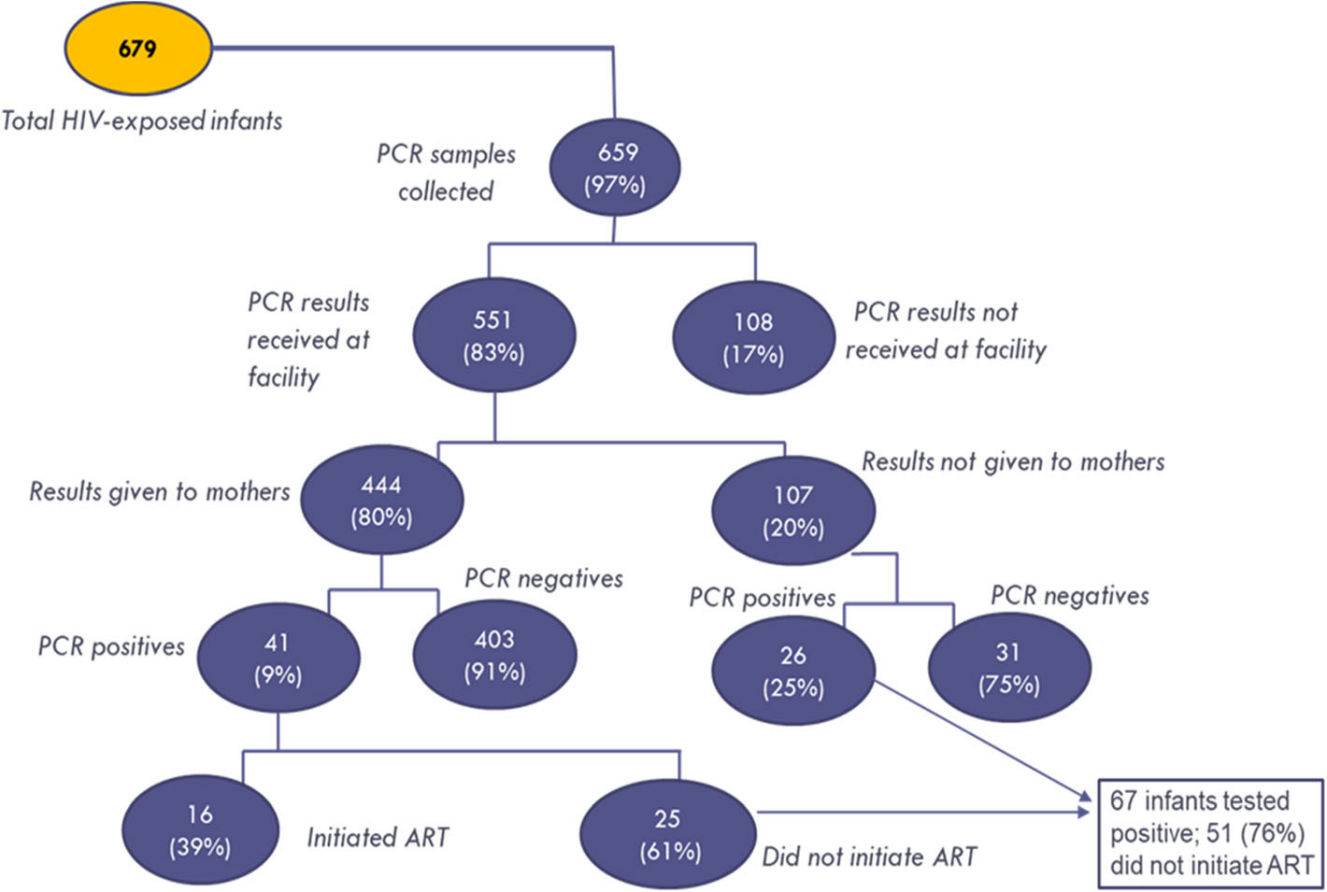
PCR results and ART initiation over three months (aggregated across six facilities)

The research team collected and aggregated data from all 6 sites to assess LTFU at each step of the cascade from CCR to ART initiation (see Fig. 2). These data were taken from clinic visit registries and the PCR samples clinic registries (see supplementary material- aggregated database). A total of 679 HIV exposed infants attended CCR care. About 97% (*n* = 659) had a blood sample taken for PCR testing, of these 83% (*n* = 551) had results received at the facility from the lab. Among these 551 results, 12% (67) were positive. But among the 80% (*n* = 444) of results actually given to mothers, 9% (*n* = 41) were positive and only 39% (*n* = 16) of those positives began ART. The 26 HIV-positive infants from the 107 test results not given to mothers did not start ART. Therefore, of 679 who should have had a PCR completed only 444 (65%) of mothers actually received results, so 35% were LTFU from PCR testing alone. And of those 41 positive infants with test results given to mothers, only 39% (16) initiated ART. Therefore, only 24% (16) of all infants who tested positive (total of 67) started ART. These data therefore indicate two key points in the cascade after arriving at CCR where HIV exposed infants are LTFU: mothers’ receipt of PCR results, and ART initiation among eligible HIV-positive infants.

### HIV-positive women’s focus group discussions

Several key themes were identified from across all six FGDs with patients. Respondents indicated that one of their greatest difficulties is the lack of support from male partners. If women tell their partners about their HIV-positive state they and their children risk being beaten or expelled from home. Because of community stigma, many women are afraid to be told that they have HIV and are ashamed to go to places where they encounter other people with HIV in facilities since they may meet neighbors or acquaintances. Fear of family disclosure and community stigma are exacerbated by the many repeat visits, long waiting periods and short consults at each stage of the treatment cascade. Each visit a mother makes, especially if the visits are longer, risks raising more suspicion from a partner. The short consultations after long waits mean mothers do not get sufficient counseling and explanation about patient navigation, pediatric HIV, treatment options, and the care cascade which heighten fear and stigma.

Other barriers identified across the groups included poor explanation at short consults and lack of understanding by mothers about next steps and follow-up appointments. This created fatigue and confusion for mothers. In all six groups, there was consensus that the long delays in delivery of laboratory results and lack of communication between patients and health professionals led to mothers often not receiving results nor understanding the consequences of a positive test, or the follow-up steps that included referrals to pediatric ART services. Mothers suggested that improved counseling, fewer visits, and active follow-up would help them be retained in care.

### Health worker focus group discussions

Several similar themes were identified in the health worker FGDs. In all the groups, health workers agreed that many women’s partners do not support HIV-positive mothers taking their children to the facility when they have no perceived reason to go. In one group, a respondent gave the example of a child completing immunization. It was difficult for the mother to explain why she needed to go to the clinic again if the male partner does not know the HIV status of the wife and child. The health workers report that there is a need to continue to involve the men in the health care of the mother and child. Several recommendations were made including more training for staff whenever there is a change in strategies, more community health workers to support services (including referral of users), and improved quality of counseling in the CPP, CCR and ART sectors. Taken together the FGD interviews indicate the need for less waiting time, fewer visits, and more support and better counseling for mothers to navigate the treatment cascade and follow-up, in part to minimize risk of disclosure to male partners.

### Health worker individual interviews

The IDIs were used to identify and diagram specific steps in the treatment cascade at each site and common steps across all sites (see Fig. 1), and elicit health worker perceptions of challenges to follow-up at those steps. The interviews underscored key findings of the health system data analysis which showed patient drop-offs at key points in the treatment cascade where longer waiting periods for mothers and transfers of patients from one service sector to another took place (e.g. CPP to CCR and CCR to ART).

## Discussion

By triangulating information from the IDIs with health facility treatment cascade data and FGD results, the research team determined the parameters for developing an intervention strategy to both streamline the treatment cascade and increase support to mothers. A sustainable and scalable intervention to reduce LTFU must rely on realistic solutions that consider the severe resource constraints in low resourced health systems serving populations with high HIV burden. The formative research approach used in this study sought to identify both the determinants of LTFU and the potential solutions that health care workers and mothers believed might be feasible and effective. The gathering of health systems data to assess the treatment cascade and analyze work and patient flow at the six sites helped identify bottlenecks and process gaps. The evaluation of data included a focus on waiting time for clinic visits, and tracking of LTFU indicators from CPP, CCR, and pediatric ART services. The qualitative interviews were essential to assess both health staff and patient experiences to help explain bottlenecks and drop-offs. Interviews with health professionals and HIV-positive mothers in CPP and CCR provided essential insights into where and why LTFU occurred at key steps in the treatment cascade, and how those challenges could be addressed in an intervention.

During the period under review, many mothers were not transferred from CPP to CCR. This transfer was critical for an early PCR test, prophylaxis, and continued maternal ART. Once in CCR, there was major variation in the waiting time for the PCR results from the sample collection to the reception of the results in the health facility in the two provinces. While some arrive on time, often many others take much longer, and this contributes to LTFU as described in the FGDs and interviews. It is important for HIV-positive children to start ART as soon as possible. The formative data showed major challenges at this stage of the cascade. Only 39% of the HIV-positive children whose mothers received the PCR result actually started ART.

The interview results revealed that waiting time for clinic visits, and lack of understanding of how to follow-up added to stigma and fear of disclosure to partners, and contributed to LTFU especially at the PCR results stage and the follow-up to ART initiation. These findings tend to corroborate results from other studies and trials in low resources contexts [10–14]. HIV testing and maternal ART initiation coverage tends be quite high, but EID and pediatric HIV care and treatment coverage are hampered by lack of patient tracking mechanisms, long waiting times for test results, and inadequate counseling and support for mothers to help them understand and navigate each step in the care cascade***.***

The formative research team solicited mothers’ and health workers’ ideas and “buy-in” for workflow modifications. The provincial health directorates were also solicited to contribute to the design of the intervention. Two core components were developed to constitute the intervention for the stepped wedge roll-out at the six sites [33]. Since the health system faced severe resource constraints, the components were designed to be modest but potentially very effective by addressing the key steps in the cascade and the concerns described by mothers and health workers. The first component deployed a patient tracking process using active follow-up of mothers with cell phone Short Message Service (SMS) messages, together with active searches and counseling by activistas to reduce LTFU from CPP to CCR. The second component centered on initiation of ART by MCH nurses in CCR, rather than referrals to separate ART services. This change would reduce number of visits, reduce LTFU from CCR to pediatric ART, and eliminate the long waits to be seen at the pediatric ART service and then to pick up ARVs at the general pharmacy. New work flow and patient flow diagrams were developed for each site.

The resources for a sustainable intervention could not address the major challenge of male partner involvement directly, but the approach sought to streamline the care cascade, with fewer visits and therefore less overall waiting time at key steps. Together with improved counseling and follow-up by nurses and activistas, the intervention could mitigate risk of disclosure to male partners, reduce fear, and reduce time to ART initiation. The intervention was initiated in 2015 through 2017 and preliminary outcome results of the intervention study have been presented elsewhere [27, 34], while final results are prepared for publication.

The formative research phase had several key strengths. The research team sought to systematically elicit the experiences and perspectives of both mothers and health workers to identify possible factors associated with LTFU and develop intervention components that both groups would consider valuable and feasible. Data were carefully gathered from health facility registries to provide a clear tracking of LTFU at each stage in the care cascade to compare to health worker and patient reports. Limitations of the study included difficulty in extracting quality data on ART initiation among infants given the lack of routine data sources and tracking in the health units. The research team was unable to conduct data quality audits to better ascertain the accuracy of routine health unit data.

## Conclusions

This formative research approach helped researchers to adjust and adapt the intervention to local needs and constraints at the six health facilities. The mixed methods approach yielded qualitative and quantitative data to triangulate and complement each other to provide a more accurate depiction of the treatment cascade across six facilities. The formative research approach was essential to adapt the intervention to the often-unpredictable context in which implementation research is conducted. These data can contribute to ongoing discussions at the MOH and among donors to modify current approaches to improve pediatric HIV care and treatment in Mozambique.

## Supplementary information

**Additional file 1.** Agregated_data_tables.

## Data Availability

The datasets generated and/or analysed during the current study are not publicly available due Bioethics Committee approval restrictions but are available from the corresponding author on reasonable request.

## Abbreviations

ANC: Antenatal care
ART: Anti-retroviral therapy
CCR: Child-at-risk care services
CPP: Post-natal care
EID: Exposed infant diagnosis
FGD: Focus group discussion
LTFU: Lost-to-follow-up
MCH: Maternal-child health
MOH: Ministry of Health
PCR: Polymerase chain reaction test
SMS: Short message service
