# Supplementary material for: HIV-exposed infant follow-up in Mozambique: formative research findings for the design of a cluster randomized controlled trial to improve testing and ART initiation
Source: BMC Health Serv Res. 2020 Mar 18;20:226. doi: 10.1186/s12913-020-5051-8 (PMC7079378; doi:10.1186/s12913-020-5051-8)
Supplement: Supplementary file 1 — Additional file 1. Agregated_data_tables. [file 12913_2020_5051_MOESM1_ESM.pdf]

Indicator- Sofala privince- CPP data	HF 1				HF 2				HF3			
	Set	Out	Nov	Total	Set	Out	Nov	Total	Set	Out	Nov	Total
No first postpartum consultation	320	231	186	**737**	155	125	62	**342**	150	36	16	202
No HIV + women at 1st visit	86	85	62	**233**	37	30	22	**89**	43	15	6	64
No HIV-exposed children referred to CCR	54	48	22	**124**	22	23	10	**55**	0	0	0	0

Indicator- Manica Province-CPP data	HF 1				HF 2				HF 3			
	Set	Out	Nov	Total	Set	Out	Nov	Total	Set	Out	Nov	Total
No first postpartum consultation	334	402	391	1127	301	273	217	791	322	464	224	1010
No HIV + women at 1st visit	21	51	15	87	16	8	5	29	20	15	11	46
No HIV-exposed children referred to CCR	22	54	25	101	10	5	5	20	0	3	0	3

Indicator-Sofala Province_Maternity data	HF 1				HF 2				HF 3			
	Set	Out	Nov	Total	Set	Out	Nov	Total	Set	Out	Nov	Total
No. of maternity deliveries	302	250	153	705	140	97	59	296	281	223	165	669
No. of HIV + women in maternity	64	70	36	170	32	18	7	57	65	41	38	144
No. of women on ART at entry	50	58	36	144	31	14	7	52	52	27	29	108
No. of exposed children who had prophylaxis	55	63	27	145	25	15	7	47	63	40	37	140

Indicator-Manica Province-Maternity data	HF 1				HF 2				HF 3			
	Set	Out	Nov	Total	Set	Out	Nov	Total	Set	Out	Nov	Total
No. of maternity deliveries	261	224	137	622	262	234	180	676	293	255	184	732
No. of HIV + women in maternity	40	25	27	92	32	25	20	77	26	22	19	67
No. of women on ART at entry	34	51	29	114	20	22	20	62	24	22	17	63

No. of exposed children who had prophylaxis	37	58	23	118	19	17	17	53	26	23	16	65

Indicador-Sofala Province-CCR data	HF1				HF2				HF3			
	Set	Out	Nov	Total	Set	Out	Nov	Total	Set	Out	Nov	Total
Nº de 1ª consultas de crianças expostas ao HIV	50	47	72	169	38	29	19	86	57	58	51	166
Nº de 1ª colheitas de PCR de crianças expostas ao HIV	56	87	69	212	31	29	20	80	53	45	42	140
Total de resultados de PCR recebidos na US	42	68	31	141	20	27	17	64	46	44	38	128
Total de resultados de PCR entregues a mae	36	58	9	103	15	24	9	48	33	30	21	84
Total de resultados de PCR positivos	4	17	8	29	3	1	1	5	6	7	7	20
Total de criancas que iniciaram TARV	2	4	3	9	1	0	0	1	1	0	0	1

Indicator-Manica Province_CCR data	HF1				HF2				HF3			
	Set	Out	Nov	Total	Set	Out	Nov	Total	Set	Out	Nov	Total
Nº de 1ª consultas de crianças expostas ao HIV	39	22	32	93	34	35	9	78	18	30	19	67
Nº de 1ª colheitas de PCR de crianças expostas ao HIV	38	22	33	93	61	33	16	110	19	23	19	61
Total de resultados de PCR recebidos na US	38	22	18	78	18	25	7	50	14	20	18	52
Total de resultados de PCR entregues a mae	35	20	16	71	15	23	4	42	14	18	16	48
Total de resultados de PCR positivos	5	0	1	6	4	3	1	8	0	2	3	5
Total de criancas que iniciaram TARV	0	0	0	0	1	2	0	3	0	1	0	1
